# Tumor Suppressor DYRK1A Effects on Proliferation and Chemoresistance of AML Cells by Downregulating c-Myc

**DOI:** 10.1371/journal.pone.0098853

**Published:** 2014-06-05

**Authors:** Qiang Liu, Na Liu, Shaolei Zang, Heng Liu, Pin Wang, Chunyan Ji, Xiulian Sun

**Affiliations:** 1 Key Lab of Otolaryngology, Qilu Hospital of Shandong University, Jinan, China; 2 Department of Hematology, Qilu Hospital of Shandong University, Jinan, China; Wayne State University, United States of America

## Abstract

Acute myeloid leukemia (AML), caused by abnormal proliferation and accumulation of hematopoietic progenitor cells, is one of the most common malignancies in adults. We reported here DYRK1A expression level was reduced in the bone marrow of adult AML patients, comparing to normal controls. Overexpression of DYRK1A inhibited the proliferation of AML cell lines by increasing the proportion of cells undergoing G0/G1 phase. We reasoned that the proliferative inhibition was due to downregulation of c-Myc by DYRK1A, through mediating its degradation. Moreover, overexpression of c-Myc markedly reversed AML cell growth inhibition induced by DYRK1A. DYRK1A also had significantly lower expression in relapsed/refractory AML patients, comparing to newly-diagnosed AML patients, which indicated the role of DYRK1A in chemoresistance of AML. Our study provided functional evidences for DYRK1A as a potential tumor suppressor in AML.

## Introduction

Acute myeloid leukemia (AML), caused by abnormal proliferation and accumulation of hematopoietic progenitor cells, is one of the most common malignancies in adults. The median age of AML patients at diagnosis is 69 [1]. Although improvement of AML treatment has been achieved during the last decades, but the five-year overall survival is still poor, especially in aged AML patients [1], [2]. The pathogenesis of AML involves variety of molecular abnormalities, mainly including activation of oncogenes and dysfunction of tumor suppressor genes [3]. Exploring new molecular mechanisms of AML is necessary to develop revolutionary therapy strategies for AML patients.

Dual specificity tyrosine-phosphorylation-regulated kinase (DYRK), which is characterized by catalyzing autophosphorylation on tyrosine residue and phosphorylation of substrates strictly on serine/threonine residues, is an evolutionarily conserved kinase family belonging to CMGC family [4]. The DYRK family, including DYRK1A, DYRK1B, DYRK1C, DYRK2, DYRK3, DYRK4A, and DYRK4B, is involved in regulating neurogenesis and cellular processes such as differentiation, proliferation, and survival [5], [6]. Recently, emerging studies have revealed their importance in development and progression of several malignancies. Taira et al. reported that DYRK2 directly phosphorylated p53 at Ser46 in osteosarcoma cells, resulting in apoptotic cell death in response to DNA damage [7]. Findings have shown that DYRK1A and DYRK2 inactivated NFATc by phosphorylation [8], [9], which increased invasive ability of breast cancer cells and drug resistance of leukemia cells [10], [11]. However, until recently, the functional role of DYRK1A in cancer is largely obscure. Interestingly, DYRK1A is localized within the Down Syndrome critical region (DSCR) on chromosome 21, and is considered to be a strong candidate gene for this genetic disorder [12], [13]. Adult with Down syndrome (DS) have a markedly decreased risk of developing cancers compared with that without DS [14], [15]. Although children with DS have a markedly increased risk of B cell precursor ALL, but T-ALL and non-megakaryoblastic myeloid leukemia are extremely rare in individuals without DS [15], [16]. We recently reported that dysregulation of DYRK1A reduced RE1 silencing factor (REST) protein stability and transcriptional activity through ubiquitination and subsequent degradation of REST protein [17]. This strongly suggests that the DYRK1A has anti-tumor effects in adult.

We report here the identification of significant lower expression of DYRK1A in adult AML patients compared to their normal controls. Overexpression of DYRK1A, by increasing the proportion of cells in the G0/G1 phase, inhibited the proliferation of AML cells. We also found that DYRK1A phosphorylated c-Myc on Ser62, priming phosphorylation on Thr58 by GSK3β and subsequent degradation. c-Myc is a critical inducer of cellular proliferation, and its abnormal expression and activation are frequently observed in most human cancers [18], [19]. Moreover, reduced AML cell growth induced by overexpression of DYRK1A was markedly reversed by c-Myc. DYRK1A also showed lower expression in relapsed/refractory patients compared to newly-diagnosed AML patents, which indicated the role of DYRK1A in drug sensitivity of AML cells. What's more, DYRK1A sensitized HL-60/ADM cell to doxorubicine. Our study contributes to reveal the molecular function of DYRK1A in pathological mechanism of AML.

## Methods

### Cell cultures and Reagents

Human myeloid leukemia cell line HEL, HL-60, NB4 and human embryonic kidney cell line HEK293 and HEK293T were purchased from the Cell Bank of the Chinese Academy of Medical Science (Shanghai, China). Human myeloid leukemia cell line HL-60/ADM were purchased from Chinese Academy of Medical Science and Peking Union Medical College(Tianjin, China). Human AML cell line HL-60, NB4, HEL, HL-60/ADM were grown in RPMI-1640 medium (Hyclone, South Logan, UT) supplemented with 10% FBS(Gibco, Gaithersburg, MD). HEK293 and HEK293T cells were cultured in high glucose Dulbecco's modified Eagle's medium (Hyclone, South Logan, UT) supplemented with 10% FBS. All cell lines were maintained at 37°C, 5% CO_2_. Primary antibodies for DYRK1A, cyclin D1, p21^Waf1/Cip1^ were purchased from CST (Beverly, MA), those for flag and β-actin were from Sigma-Aldrich (St Louis, MO), those for c-Myc was from Santa Cruz (Santa Cruz, CA), those for c-Myc pThr58 and pSer62 were from Immunoway (Newark, DE). All secondary antibodies were obtained from Jackson Immuno Research (West Grove, PA).

### Clinical samples

Bone marrow samples of 55 initially diagnosed and 16 relapsed/refractory adult AML patients and 24 healthy donors were obtained after informed consent at the Qilu Hospital, Shandong University from May 2011 to December 2012. Mononuclear cells were prepared using Ficoll-Hypaque (Sigma–Aldrich, St Louis, MO), according to the manufacturer's protocol. All the study protocols involved with patients and healthy donors were approved by the Medical Ethics Committee of Qilu Hospital of Shandong University, Jinan, China, and written informed consents were obtained from all patients and healthy donors. The detailed clinical information of the 71 patients is presented in Table 1.

**Table 1:** Characteristics of the 71 AML patients.

	No. of patients	
	Newly-diagnosed	Relapsed/refractory
No. of patients	55	16
Sex		
male	31	9
female	24	7
Age: median (range)	48 (16–77)	42.5 (17–61)
FAB Classification		
M0	2	0
M1	2	0
M2	6	5
M3	13	5
M4	15	4
M5	14	1
M6	3	1
Hemoglobin (g/l): median (range)	75 (33–136)	70.1 (48–135)
WBC (x109/l): median (range)	6.65 (1.18–169.5)	9.01 (0.88–146.1)
Neutrophils (%): median (range)	3.86 (0.09–129.9)	5.88 (0.16–122.71)

Abbreviations: FAB, French-American-British classification; WBC, white blood cells; PLT, platelets.

### Plasmid construction and lentivirus packaging

Human *DYRK1A* cDNA was generated as described previously [17]. Human *c-Myc* CDS was amplified from NB4 cells by RT-PCR using the primer pair: 5′- GAAGATCTCTGGA TTTTTTTCGGGTAGTGG -3′ and 5′- CGGAATTCTTACGCACAAGAGTTCCGTAG -3′. PCR products were cloned into eukaryotic expression vector pEGFP-C1. *DYRK1A* and *c-Myc* were subcloned into lentiviral vectors pWPXL. GV248-si-*c-Myc* lentiviral vectors were purchased from Genechem(Shanghai, China). 4×10^6^ HEK293T cells were plated 1 day before transfection in 10 cm dish and 20 µg DNA were transfected per dish using the calcium phosphate precipitate method. The supernatant containing viral particles were collected at 48 and 72 hr after transfection, filtered through a 0.45 µm filter, concentrated by 100KD centrifugal filter(Millipore, Billerica, MA) and then stored at −80°C.

### MTT assay

Cells (3×10^3^) were seeded into 96-well culture plates. When measuring, cells were incubated with 10 µL MTT (5 mg/mL) at 37°C for 4 hrs, and then 100 µL 10% SDS(PH 4.0) was added to each well and incubated over night. The absorbance was measured at 570 nm. Each experiment was repeated at least three times.

### Cell cycle analysis

Cells were harvested and washed twice in PBS, then fixed in 75% alcohol over night at 4°C. After washed in cold PBS thrice, cells were resuspended in 1 mL PBS with 40 µg PI and 100 µg RNase A (Sigma-Aldrich, St Louis, MO) and incubated for 30 min at 37°C. Samples were then analyzed by FACS(Beckman, CA).

### Apoptosis assay

The apoptosis assay was performed using the Annexin V-PE/7-AAD apoptosis detection kit(Ebioscience, San Diego, CA), according to the manufacturer's instructions. Fluorescence of at least 10,000 cells was collected by FACS(Beckman, CA). to determine the percentage of apoptotic cells.

### Quantitative real-time RT-PCR

TRIzol reagent (Invitrogen, Carlsbad, CA) was applied to extract total RNA from cells or patient samples. Reverse transcription was performed using M-MLV reverse transcriptase cDNA Synthesis Kit(Takara, Japan). Real-time RT-PCR was carried out on ABI 7900HT Fast Real-Time PCR System(Foster City, CA) with SYBR-Green PCR Master Mix (Toyobo, Japan) as described previously[20]. Melting curves analysis and agarose gel electrophoresis were applied to guarantee the specificity of amplification. A comparative CT method (2^−ΔΔCT^) was used to analyze the gene expression level. *β-actin* or *GAPDH* were used as the internal control. The primers for real time quantitative were as follows: *DYRK1A*-F 5'-GCAATTTCCTGCTCCTCTTG-3'; *DYRK1A*-R 5'-TTACCCAAGGCTTGTTGTCC-3'; *c-Myc*-F 5′-TCAAGAGGTGCCACGTCTCC-3′; *c-Myc*-R, 5′-TCTTGGCAGCAGGATAGTCCTT-3′; *cyclin D1*-F5′-GCTGCGAAGTGGAAACCATC-3′; *cyclin D1*-R 5′-CCTCCTTCTGCACACATTTGAA-3′; *p21^Waf1/Cip1^*-F 5′-TGTCCGTCAGAACCCATGC-3′; *p21^Waf1/Cip1^*-R 5′-AAAGTCGAAGTTCCATCGCTC-3′; *CDK2*-F 5′-CCAGGAGTTACTTCTATGCCTGA-3′; *CDK2*-R 5′-TTCATCCAGGGGAGGTACAAC-3′; *β-actin*-F 5′-CACTGTGTTGGCGTACAGGT-3′; *β-actin*-R 5′-TCATCACCATTGGCAATGAG-3′; *GAPDH*-F 5′- TGTGGGCATCAATGGATTTGG-3′; *GAPDH*-R 5′-ACACCATGTATTCCGGGTCAAT-3′.

### Western Blot

For western blot analysis, cells were harvested and washed by ice-cold PBS and lysed by sonication in RIPA buffer (150 mM NaCl, 50 mM Tris-HCl, 1% Triton X-100, 2% SDS, and 1% sodium deoxycholate) containing protease inhibitor cocktail (Roche, Indianapolis, IN). Bio-Rad Dc protein assay kit (Bio-Rad, Richmond, CA) was used to measure the concentration of protein samples. Samples were denatured at 95°C with 4× SDS sample buffer for 5 min in metal bath and separated on 10% glycine SDS-PAGE gel. Membranes were blocked in 3% non-fat milk for 1 hous and incubated in primary antibodies with 1∶1000 dilution (TBS with 0.1% Tween-20, 1% goat serum) overnight. After secondary antibody incubation, signals were detected and analyzed by the Li-Cor Odyssey imaging system.

### CHX chase assay

c-Myc and DYRK1A or control vectors were co-transfected into HEK293 cells. 36 hrs after transfection, cells were treated with 50 µg/mL cycloheximide (Sigma-Aldrich, St Louis, MO) for 0, 1 and 2 hrs. Cells were then harvested and analyzed by western blot.

### Co-immunoprecipitation

For co-immunoprecipitation, the cells were lysed in 1 mL 1% NP-40 lysis buffer(1% NP-40, 50 mM Tris-base, 150 mM NaCl). Cell lysates were gently shaken with 100 µL CL-4B (Sigma-Aldrich, St Louis, MO) at 4°C for 1 hr, then centrifuged at 15000 r/min 4°C for 15 min. The immunoprecipitation antibody recruited with 20 µL protein A/G (Santa Cruz Biotechnology, Santa Cruz, CA) were mixed with the supernatant and shaken at 4°C overnight. The protein-agarose beads mixture was washed once by 1% NP-40 lysis buffer and twice by ice-cold PBS, boiled with 4×SDS sample buffer, then analyzed on 10% glycine SDS-PAGE gel.

### Data Analysis

All the experiments were repeated at least three times. For immunoblotting, one representative picture was shown, while quantifications were calculated from at least three independent experiments. The values represented means ± S.E. The data were evaluated for statistical significance by analysis of variance or Student's t test analysis. For patient samples, *DYRK1A* mRNA level was presented quantitatively as median. The differences in the newlydiagnosed, relapsed/refractory patients and normal controls were performed using a one-way ANOVA test. All statistical analysis were processed by SPSS 17.0 software.

## Results

### Aberrant expression profile of DYRK1A in adult AML patients

**Figure 1:**
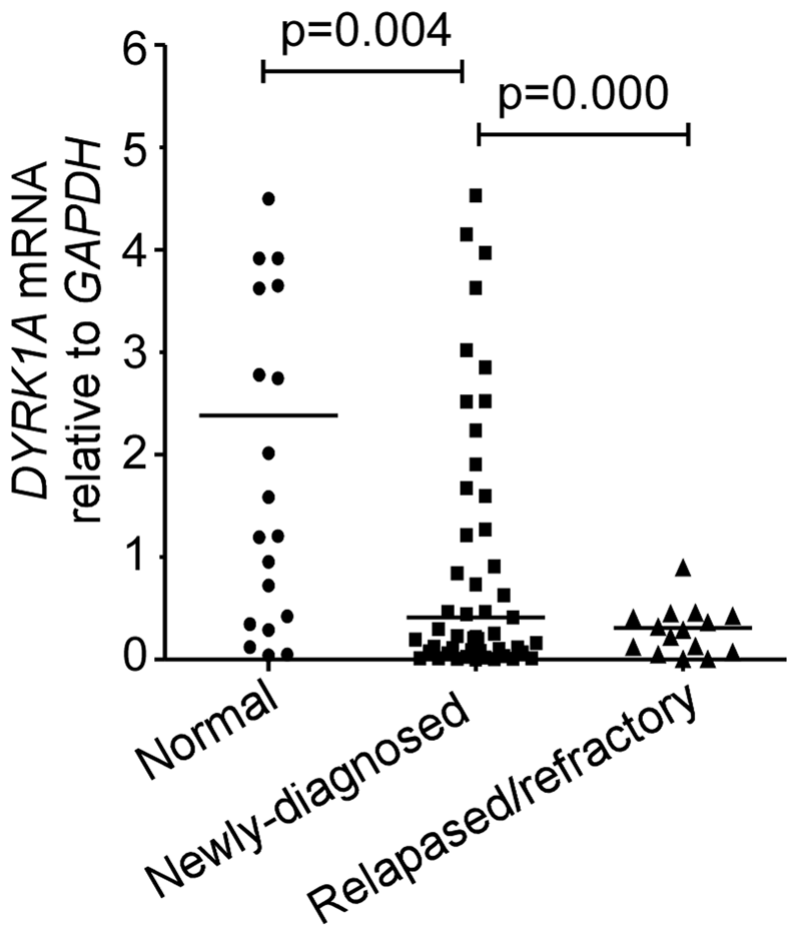
Real-time RT-PCR of *DYRK1A* mRNA level of in 55 newly diagnosed adult AML patients, 16 relapsed/refractory adult AML patients and 24 normal controls. *GAPDH* was used as internal control. Solid points indicate individual values and horizontal lines represent medians. The differences in copy number of each gene in the newly diagnosed, relapsed/refractory patients and normal controls were performed using a one-way ANOVA test.

Relative *DYRK1A* mRNA levels of the newly diagnosed adult AML patients and healthy controls were measured by real-time RT-PCR. In spite of the wide range of individual values, median level of *DYRK1A* was significantly reduced in the AML patients compared with the normal controls (P = 0.004) (Figure 1). The attenuated level of *DYRK1A* was also observed in relapsed/refractory AML patients compared with untreated AML patients (p = 0.000) (Figure 1).

### DYRK1A suppresses proliferation of AML cells by extending the G0/G1 phase

**Figure 2:**
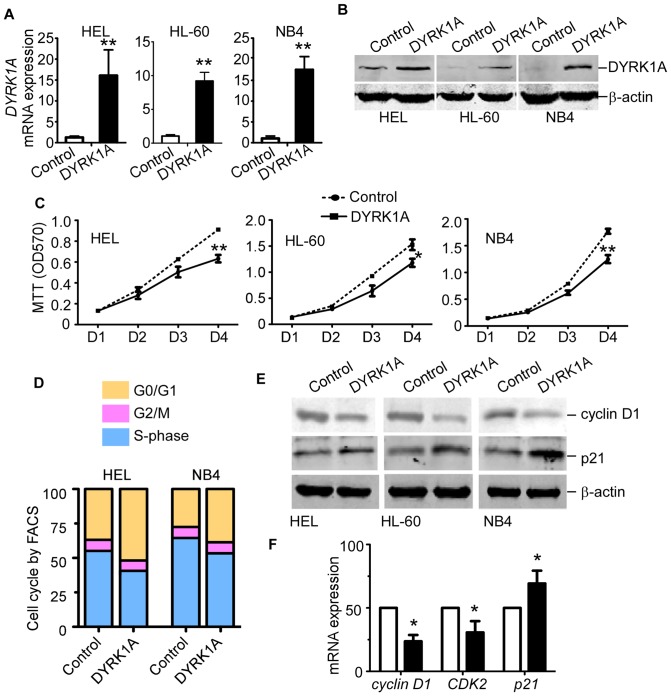
DYRK1A suppressed proliferation of AML cells by extending the G0/G1 phase. HEL, HL-60 and NB4 cells were infected with DYRK1A lentiviral particles(DYRK1A) or negative control (Control) for 72 hrs. (A) Real-time RT-PCR was performed to assess the increment of *DYRK1A* mRNA level. *DYRK1A* mRNA levels were normalized to *β-actin*. The results present the fold-increase relative to control. Data represents mean ± S.E. from three independent experiments. **P<0.01, (B) Western Blot was used to detect the DYRK1A protein level increment. β-actin was used as loading control. Experiments were repeated three times, and one representative figure was shown, (C) After being infected with DYRK1A lentiviral particles for 72 hrs, HEL, HL-60 and NB4 cell proliferation were measured by MTT assay. The values represent the means ± S.E. (n = 3). *P<0.05, **P<0.01, (D) After being infected with DYRK1A lentiviral particles for 72 hrs, HEL and NB4 cells were stained with PI and analyzed for their DNA content using FACS Calibur. Results shown are representative of at least three independent experiments. (E) Western blot of cell cycle regulators cyclin D1, CDK2 and p21 in HEL, HL-60 and NB4 cells. β-actin was used as loading control. Results shown are representative of at least three independent experiments. (F) Real-time RT-PCR detected *cyclin D1*, *CDK2* and *p21* mRNA levels in HEL cells after being infected with DYRK1A lentiviral particles for 72 hrs. *β-actin* was used as control. The values represent the means ± S.E. (n = 3). *P<0.05.

To observe the effects of DYRK1A on the growth of HEL, HL-60 and NB4 cells, cell proliferation assays were performed after infection with DYRK1A lentiviral particles or negative control for 72 hrs. RT-PCR and western blot assay showed that DYRK1A lentiviral particles could efficiently increase *DYRK1A* mRNA and protein levels in AML cells (Figure 2 A and B). As shown in Figure 2C, overexpression of DYRK1A led to consistently reduced cell growth compared with negative control in HEL, HL-60 and NB4 cells. We next questioned whether inhibited proliferation was induced through the arrest of cell cycle. As shown in Figure 2D, overexpression of DYRK1A significantly increased the ratio of cells in G0/G1 phase while concomitantly reduced the ratio of cells undergoing S phase, To exclude the influence of cell apoptosis, cells were stained with annexin V-PE/7-AAD and analyzed by flow cytometry. We did not find significant change of cell apoptosis between DYRK1A overexpression and control cells (data not shown). To further identified the genes that were responsible for the cell cycle arrest after DYRK1A overexpression, representative cell cycle regulators were studied. p21 was increased, while cyclin D1 and CDK2 were down-regulated by DYRK1A overexpression in AML cells (Figure 2E and F). These results provided evidences that DRYK1A suppressed proliferation of AML cells by extending the G0/G1 phase.

### c-Myc expression is down-regulated by DYRK1A

**Figure 3:**
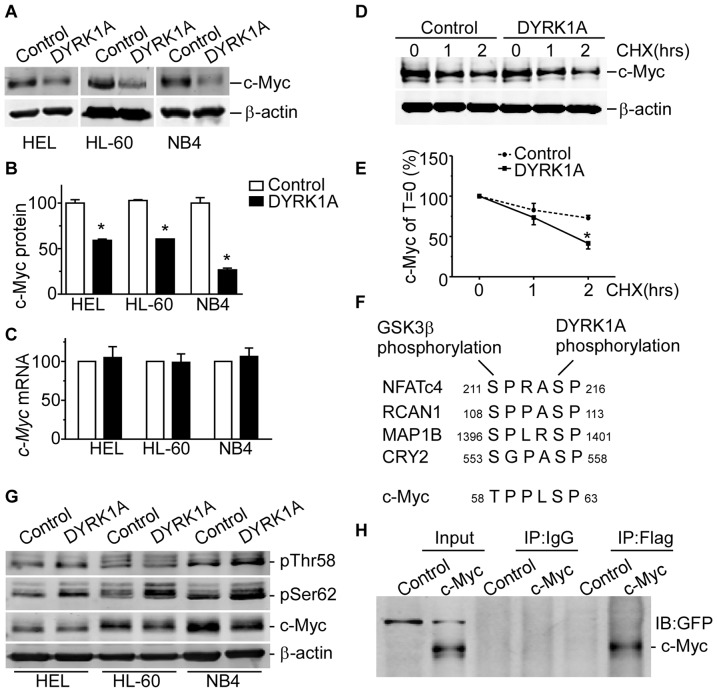
c-Myc expression is down-regulated by DYRK1A. (A and B) HEL, HL-60 and NB4 cells were infected with DYRK1A lentiviral particles or negative control for 72 hrs. Western blot showed that c-Myc were downregulated in AML cell lines, compared with negative control, respectively. β-actin was used as loading control. Results shown are representative of at least three independent experiments. The values represent the means ± S.E. (n = 3). *P<0.05. (C)HEL, HL-60 and NB4 cells were infected with DYRK1A lentiviral particles or negative control for 72 hrs. Real time RT-PCR showed the *c-Myc* mRNA levels in AML cell lines. The values represent the means ± S.E. (n = 3). *P<0.05. (D and E) HEK293 cells co-transfected with pEGFP-*c-Myc* vector and pCMV6 or pCMV6-*DYRK1A* were chased with 50 µg/mL cycloheximide(CHX) for 1 and 2 hrs. c-Myc expression was detected by anti-GFP antibody. β-actin was used as loading control. The values represent the means ± S.E.(n = 3). *P<0.05. (F) Sequence alignments around phosphorylation residues of GSK3β and DYRK1A substrates, as well as c-Myc. (G) HEL, HL-60 and NB4 cells were infected with DYRK1A lentiviral particles or negative control for 72 hrs. Cells were lysed for western blot. Anti-c-Myc, anti-c-Myc(pThr58), anti-c-Myc(pSer62) antibodies were applied for analysis.β-actin was used as loading control. (H) HEK293 cells co-transfected with pCMV6-*DYRK1A* and pEGFP-c1 or pEGFP-C1-*c-Myc* were lysed and immunoprecipitated with anti-flag antibody, anti-GFP antibody was used in immunoblotting.

c-Myc is a critical inducer of cellular proliferation, and its dysregulation is frequently observed in most human cancers. To determine whether DYRK1A can regulate c-Myc, we measured c-Myc protein and mRNA level in DYRK1A overexpressed AML cells. We found that c-Myc protein was downregulated by DYRK1A overexpression in HEL, HL-60 and NB4 cells (Figure 3A). Western blot quantification showed that c-Myc protein levels were markedly reduced by DYRK1A overexpression by 40.90±2.43%, 39.46±0.98% and 73.29±3.37% of controls in HEL, HL-60 and NB4 cells, respectively (P<0.01)(Figure 3B), while DYRK1A could not significantly change *c-Myc* mRNA level(Figure 3C), indicating that the reduction of c-Myc was due to post-translational regulation. To further investigate whether c-Myc reduction is due to decreased protein stability, HEK293 cells were co-transfected with c-Myc and DYRK1A or control and cycloheximide chase assay was performed to evaluate the degradation rate of c-Myc. Western blot clearly showed that the degradation of c-Myc protein was accelerated in the presence of DYRK1A overexpression (Figure 3D and E). Phosphorylation on Thr58 residue is necessary for binding between E3 ligase SCF^Fbw7^ and c-Myc, and subsequent ubiquitination and proteolysis [21]. Phosphorylation on c-Myc Thr58 is mainly catalyzed by GSK3β [21], which targets the first Serine or Threonine of the conserved sequence S/T PxxSP, primed by the phosphorylation of the second Serine. DYRK1A has been reported several times to act as a phosphorylation partner of GSK3β [9], [22], [23], [24], which are aligned in Figure 3F. From Figure 3F we can see that c-Myc resembles a similar sequence from Thr58 to Ser62 with other GSK3β substrates. We speculate that DYRK1A phosphorylates on c-Myc Ser62, priming the phosphorylation of Thr58 by GSK3β and its degradation by ubiquitin-proteasome system. Three days after infection with lentiviral particles, HEL, HL-60 and NB4 cells were lysed for Western Blot. Our results showed overexpression of DYRK1A downregulated c-Myc protein level, consistent with our previous studies. Phosphorylation status of c-Myc Ser62 and Thr58 was significantly increased by DYRK1A overexpression (Figure 3G). To further assess whether there is a direct interaction between DYRK1A and c-Myc in mammalian cells, HEK293 cells were co-transfected with pCMV6-*DYRK1A* and pEGFP-c1 or pEGFP-C1-*c-Myc*. c-Myc was detected by anti-GFP antibody in co-immunoprecipitation assay pulling down with anti-flag antibody, indicating an direct interaction between DYRK1A and c-Myc (Figure 3H). These results strongly demonstrated that DYRK1A reduced c-Myc level via promoting its degradation.

### DYRK1A suppresses proliferation of AML cells through downregulating c-Myc

**Figure 4:**
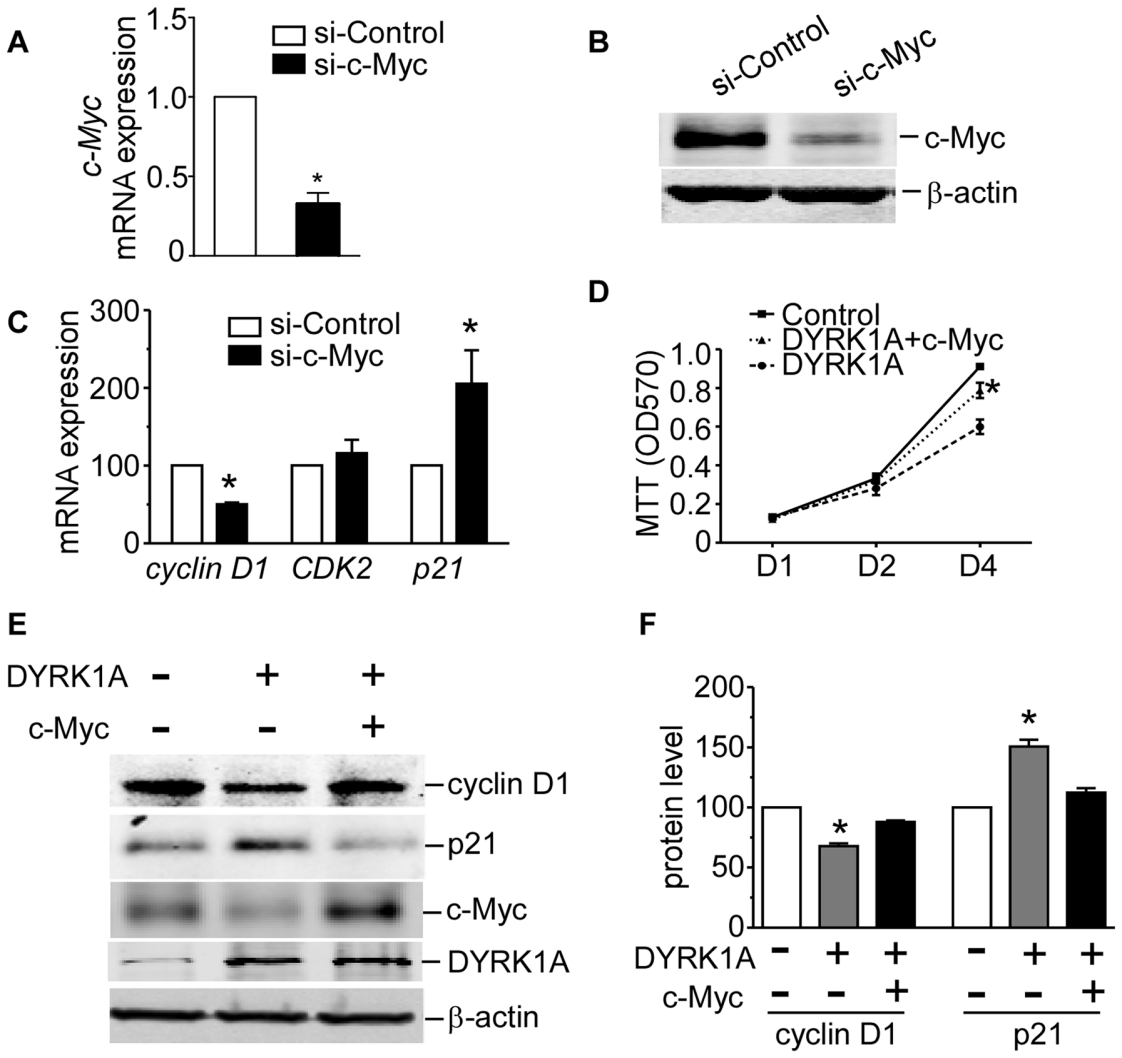
DYRK1A suppresses proliferation of AML cells through downregulating c-Myc. (A and B) HEL cells were infected with pSV248-sic-Myc lentiviral particles(si-c-Myc) or negative control(si-Control) for 72 hrs. Real-time RT-PCR and western blot was used to validate the siRNA effect on c-Myc expression. For real-time PCR, data were calculated from 3 experiments. Values represent the means ± S.E. (n = 3). *P<0.05. For western blot, one representative figure from three experiments was shown. (C) HEL cells were infected with pSV248-si*c-Myc* lentiviral particles(si-*c-Myc*) or negative control(si-Control) for 72 hrs. Real-time RT-PCR detected *cyclin D1*, *CDK2* and *p21* mRNA levels in HEL cells. *β-actin* was used as internal control. The values represent the means ± S.E. (n = 3). *P<0.05. (D)HEL cells were infected with DYRK1A lentiviral particles(DYRK1A) or negative control(Control) and c-Myc lentiviral particles(c-Myc) for 72 hrs. After infection, cells were subjected to the MTT assay. The values represent the means ± S.E.(n = 3). *P<0.05(DYRK1A+c-Myc vs DYRK1A) (E and F) HEL cells were infected with DYRK1A lentiviral particles(DYRK1A) or negative control(Control) and c-Myc lentiviral particles(c-Myc) for 72 hrs. Cell cycle regulators cyclin D1 and p21 were detected by western blot. Results shown are representative of at least three independent experiments. The values represent the means ± S.E. (n = 3). *P<0.05.

To determine whether c-Myc affects proliferation of AML cells, we first silenced c-Myc (Figure 4A and B) and found significant reduction of *cyclin D1* mRNA level by 49.53±2.43% (p = 0.006), and marked elevation of *p21* mRNA level by 105.36±42.94% (p = 0.015), to negative controls, respectively (Figure 4C). Next, we confirmed whether cell growth inhibition induced by DYRK1A overexpression was reversed by upregulating c-Myc expression. We found reduced AML cells growth by overexpression of DYRK1A was markedly attenuated by expression of c-Myc (Figure 4D). This was further supported by changes of cell cycle markers. We found that overexpressing of c-Myc reversed the downregulation of cyclin D1 and upregulation of p21 caused by DYRK1A overexpression (Figure 4E and F). Our studies revealed DYRK1A suppressed AML cells proliferation through downregulation of c-Myc.

### Upregulation of DYRK1A increases drug sensitivity of HL-60/ADM

**Figure 5:**
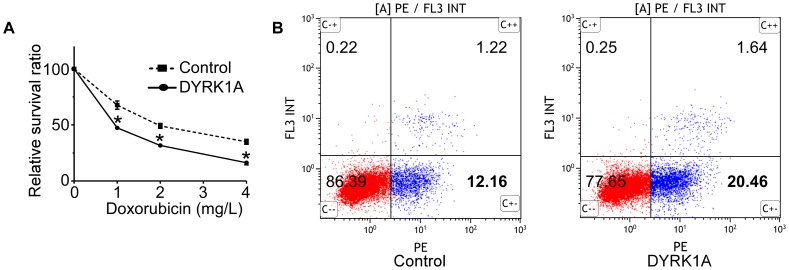
Upregulation of DYRK1A increases drug sensitivity of HL-60/ADM. (A) HL-60/ADM cells infected with DYRK1A lentiviral particles(DYRK1A) or negative control(Control) were treated with gradient dilutions of doxorubicin for 72 hrs (0, 1, 2, 4 mg/L). Cell viability was measured by MTT assays. Results represent the mean ± S.E. from three independent experiments. *P<0.05. (B) HL-60/ADM cells infected with DYRK1A lentiviral particles(DYRK1A) or negative control(Control) were cultured in 0.5 mg/L doxorubicin for 48 hrs. Apoptosis was quantified by flow cytometry with dual staining of Annexin V-PE and 7-AAD. Dot plots of representative experiments showing apoptotic cell detection after 48 hrs of treatment with doxorubicin. Percentages of early (AV+7-AAD-) and late (AV+7-AAD+) apoptotic cells were calculated.

As shown in Figure 1, the reduced level of *DYRK1A* was observed in relapsed/refractory AML patients compared with untreated AML patients, suggesting that DYRK1A could play an important role in chemoresistance of AML patients. We then investigated the effect of DYRK1A on drug sensitivity of HL-60/ADM, a multidrug resistant leukemia cell line. As shown in Figure 5A, DYRK1A sensitized HL-60/ADM cell to doxorubicine. IC50 values(drug concentration leads to 50% decrease of cell viability), is an intelligible indicator of drug sensitivity. IC50 value of HL-60/ADM cells transfected with DYRK1A was significantly lower than that of control group. The IC50 values of HL-60/ADM-DYRK1A and HL60/ADM control were 0.9389±0.063,2.3144±0.58299, respectively (P<0.01). Transfected or control HL-60/ADM cells were treated with 0.5 mg/L doxorubicin for 48 h. After labeled with Annexin V-PE/7-AAD, cells were analyzed by flow cytometry. HL-60/ADM cells overexpressing DYRK1A showed a higher percentage of apoptosis than control (Figure 5B). These results demonstrated that upregulation of DYRK1A promoted apoptosis induced by doxorubicin in HL-60/ADM.

## Discussion

In this study, we found that DYRK1A mRNA level was reduced in newly-diagnosed adult AML patients comparing to normal controls and overexpression of DYRK1A markedly inhibited proliferation of in AML cell lines. What's more, DYRK1A has been recently reported to mediate inactivity of several oncogenic proteins including NFATc [8], [9], cyclin D1 [25], and NOTCH [26]. These findings suggest the important role of DYRK1A as a tumor suppressor in adult AML.

Down syndrome (DS) associated neurogenesis defects are characterized by slowed proliferation of neural precursor cells. DYRK1A, as an important triggering protein in the pathogenesis of DS, was reported to impair G0/G1-S phase transition and alter neural precursor cell proliferation [27]. Litovchick et al. found DYRK1A inhibited various cells proliferation, including T98G brain glioblastoma cells, U-2 OS osteosarcoma cells and SW 1990 pancreatic adenocarcinoma cells [28]. Chen et al. reported DYRK1A increased G1 duration in BJ-5ta foreskin fibroblast cell line [25]. In our study, we found overexpression of DYRK1A inhibited the proliferation of AML cell lines by cell cycle arrest. Our results confirmed the previous study about the proliferation regulating functions of DYRK1A.

However, the mechanism of how DYRK1A regulates cell proliferation is still unclear. Litovchick et al. reported that DYRK1A specifically phosphorylated LIN52 on Ser28, which was required for DREAM complex assembly, repressing the expression of cell cycle-dependent genes during quiescence [28]. Chen et al. reported DYRK1A increased G1 duration via phosphorylation and subsequent degradation of cyclin D1 as well as stabilization of p21 [25]. In our study, we found that DYRK1A regulated cell cycle related genes, cyclin D1 and p21, at mRNA level, which prompted a new molecular mechanism.

The oncogene c-Myc plays an important role in tumorgenesis, e.g. lymphoma [29] and leukemia [30]. Over the past decades, however, a large body of research showed c-Myc activity were essential for cell cycle progression. Mateyak et al. reported c-Myc null cells ceased to proliferate and exited the cell cycle [31]. c-Myc played a central role in promoting G1 to S phase cell cycle transition. The possible mechanism was through regulating cyclins and CKIs [32]. Other studies reported similar results. Daksis et al. found the activation of c-Myc was sufficient to induce cyclin D1 mRNA as well as protein expression [33]. Seoane et al. reported c-Myc attenuated p21 expression by directly binding its promoter [34]. All these studies have proved that c-Myc acts as an upregulator of cyclin D1 and a repressor of p21 at transcription level, promoting G0/G1 to S phase transition. Here we found overexpression of DYRK1A downregulated c-Myc protein other than mRNA, indicating DYRK1A regulates c-Myc at post-transcription level. Moreover, DYRK1A had been reported to impact protein stability through phosphorylation, e.g. REST [17], RCAN1 [22] and CRY2 [24]. Here we confirmed when co-expressed with DYRK1A in AML cells, c-Myc was accelerated for degradation when chased with cycloheximide. These studies suggest DYRK1A regulates cell cycle by influencing c-Myc degradation and subsequently cyclin D1 and p21.

We also observed downregulated *CDK2* mRNA induced by overexpression of DYRK1A, however, CDK2 is not a downstream target of c-Myc, indicating other mechanisms were involved in regulating *CDK2* mRNA level. *CDK2* mRNA expression is induced by MITF [35] and repressed by p53 and IRF1 [36]. MITF drives most of the melanocytic markers, and interestingly, *DYRK1A* mRNA is downregulated in melanoma cell lines [37]. DYRK1A phosphorylates p53 at Ser 15 and increases its transcriptional activity in H19-7 cells [27]. In Figure 2, we showed when DYRK1A was overexpressed in HEL cell, *CDK2* mRNA level was downregulated, which was consistent with these studies.

In Down Syndrome, DYRK1A overexpression is due to the extra copy of chromosome 21(or part of chromosome 21), which is known as gene dosage effect. But in most cases, *DYRK1A* gene dosage effect does not exist in pathological and physiological processes, whereas *DYRK1A* mRNA abundance differs strikingly in various tissues and time points of differentiation and proliferation. DYRK1A was found to be downregulated in melanoma [37], and overexpressed in the activation of T-cells [38]. In neural progenitor cells, *DYRK1A* expression was restricted to M-G1 phase [39]. These studies indicate that DYRK1A is tightly controlled at transcriptional level, however, the mechanisms are not clear. AP4 was reported to be strongly expressed in colorectal carcinomas [40] and repress the transcription of *DYRK1A* in coordinate with geminin [41]. E2F1 was shown to bind to the promoter of *DYRK1A* and activate its transcription [42], though E2F1 was reported to promote myeloid cell cycle progression and block granulocyte differentiation [43]. Youngkyun Lee et al. reported NFAFc1 regulated *DYRK1A* in a feedback loop [44]. Our study showed REST could directly bound to *DYRK1A* promoter, initiating the transcription [17], and previous research proved REST was an tumor suppressor in breast cancer cells [45], [46]. Despite these studies, the mechanism in AML of downregulation of *DYRK1A* mRNA level is still unclear. More studies about the functions of these factors are necessary and researchers should take effort to discover new factors regulating *DYRK1A* transcription in AML.

In summary, we found that DYRK1A level was decreased in adult AML patients and overexpression of DYRK1A caused cell cycle arrest and proliferation inhibition in AML cells. We reasoned that the proliferation inhibition could be due to the role of DYRK1A in downregulation of c-Myc by mediating its degradation. Our results clearly show DYRK1A acts as a potential tumor suppressor in AML. Our findings may also present new opportunities for exploitation in novel treatment strategies in AML.
